# National burden of gambling in Japan: an estimation from an online-based cross-sectional investigation and national epidemiological survey

**DOI:** 10.1186/s12889-024-19197-z

**Published:** 2024-06-26

**Authors:** Chiyoung Hwang, Ryuhei So, Nozomu Hashimoto, Toshiaki Baba, Sachio Matsushita, Matthew Browne, Toshiya Murai, Norio Watanabe, Naoko Takiguchi

**Affiliations:** 1 Department of Psychiatric and Mental Health Nursing, Graduate School of Nursing, Mukogawa Women’s University, 6-46 Ikebiraki-cho, Nishinomiya-city, 663-8558 Hyogo Japan; 2 Department of Health Promotion and Human Behavior, School of Public Health, Kyoto University, Yoshida-Konoe-cho, Sakyo-ku, Kyoto-city, Kyoto, 606-8501 Japan; 3 Department of Psychiatry, Okayama Psychiatric Medical Center, 3-16 Shikatahommachi, Kita-ku, Okayama-city, Okayama, 700-0915 Japan; 4 Scientific Research WorkS Peer Support Group (SRWS-PSG), 1-7-7-2302 Koraibashi, Chuo-ku, Osaka-city, Osaka, 541-0043 Japan; 5 Bureau of International Health Cooperation, National Center for Global Health and Medicine, 1-21-1 Toyama, Shinjuku-ku, Tokyo, 162-8655 Japan; 6 National Hospital Organization Kurihama Medical and Addiction Center, 5-3-1 Nobi, Yokosuka-city, 239-0841 Kanagawa Japan; 7 School of Health, Medical and Applied Sciences, Central Queensland University, 6 University Drive, Branyan, QLD 4670 Australia; 8 Department of Psychiatry, Graduate School of Medicine, Kyoto University, Yoshida-Konoe-cho, Sakyo-ku, Kyoto-city, Kyoto, 606-8501 Japan; 9 Department of Psychiatry, Soseikai General Hospital, 101 Shimotobahirosacho, Fushimi-ku, Kyoto-city, Kyoto, 612-8473 Japan; 10 Department of Sociology, Otani University, Koyama-Kamifusacho, Kita-ku, Kyoto-city, Kyoto, 603-8143 Japan

**Keywords:** Gambling, Gambling harms, Prevention paradox, Public health, Online survey

## Abstract

**Background:**

Gambling is a popular leisure activity in many countries, often expected to boost regional economies. Nevertheless, its negative impacts remain a significant concern. Gambling disorder is recognized as the most severe consequence; however, even non- or low-risk gamblers may also face negative impacts. This study aimed to estimate the number of Japanese gamblers experiencing gambling-related harm (GRH) and its distribution across six life domains, financial, relational, emotional, health, social and other aspects, based on the severity of their problem gambling risk.

**Methods:**

This cross-sectional study relied on an online survey conducted between August 5 and 11, 2020. Participants aged 20 years and above, who engaged in gambling during 2019 were recruited via a market research company. The survey assessed the prevalence of GRH 72 items among four gambler risk groups (non-problem, low-, moderate-, and high-risk), as categorized by the Problem Gambling Severity Index. The data was adjusted for population weighting using representative national survey data: *the 2017 Comprehensive Survey of Living Conditions* and *the 2017 Epidemiological Survey on Gambling Addictions*.

**Results:**

Out of the 28,016 individuals invited to the survey, 6,124 participated in the screening, 3,113 in the main survey, and 3,063 provided valid responses. After adjusting the survey data, it was estimated that 39.0 million (30.8%) of Japan’s 126.8 million citizens gambled in 2019. Among them, 4.44 million (11.4%) experienced financial harm, 2.70 million (6.9%) health harm, 2.54 million (6.5%) emotional harm, 1.31 million (3.4%) work/study harm, 1.28 million (3.3%) relationship harm, and 0.46 million (1.2%) other harm. Although high-risk gamblers experienced severe harm at the individual level, over 60% of gamblers who experienced GRHs were non- and low-risk gamblers, with the exception of other harm, at the population level.

**Conclusions:**

The study highlighted the prevention paradox of gambling in Japan. While national gambling policies primarily focus on the prevention and intervention for high-risk gamblers, a more effective approach would involve minimizing GRH across the entire population.

**Supplementary Information:**

The online version contains supplementary material available at 10.1186/s12889-024-19197-z.

## Background

Gambling is a popular leisure activity in many countries, contributing to increased corporate profits and tax revenue [1, 2]. In Japan, various gambling options exist, including government-managed activities such as horse racing, motorboat racing, and bicycle racing, alongside private operations such as Pachinko and Pachi-slots (electronic gaming machines: EGMs). EGMs are internationally recognized as forms of gambling [3]; however, Japanese law categorizes them as leisure activities [4] owing to a legal loophole allowing players to exchange tokens for money outside parlors. These parlors are widespread in Japan and house approximately 60% of global EGMs [5]. Its market size of 14.6 trillion yen surpassed the 8.9 trillion yen from government gambling in 2022 [6]. Furthermore, the Japanese government is promoting integrated resort facilities with casinos to stimulate regional economies [7]. Osaka is set to open Japan’s first casino in 2030 [8].

While gambling is a widely enjoyed leisure activity with economic benefits, concerns about its harm persist. The most severe harm is generally recognized in the form of gambling disorder, an addictive mental disorder experienced by high-risk individuals of problem gambling. This disorder is defined by compulsive gambling behaviors that lead to significant personal distress and impairment, including a loss of control and continued gambling despite adverse consequences [9]. The prevalence of problem gamblers in the past year varies between 0.12 and 5.8% worldwide [10] and 1.6% in Japan [11]. Although this prevalence is one of the critical indicators of gambling harm, importantly, even non-disordered gamblers (non- or low-risk individuals of problem gambling) can experience negative consequences due to gambling, such as financial loss, reduced well-being, and deteriorating relationships. Recently, the concept of ‘gambling-related harms’ (GRHs) has gained international recognition; this encompasses a broader range of harms affecting financial, relationship, emotional, health, and social aspects experienced by individual gamblers, their close associates, and communities [12].

GRH has been assessed in several countries. Studies in Australia [13, 14], New Zealand [15], and Finland [16] reported that over 60% of GRHs at the population level are attributed to non- and low-risk gamblers. In contrast, high-risk gamblers experience many and more severe harms at an individual level [16]. This phenomenon, whereby the more prevalent lower-risk group contributes the majority of impact at the population level, is known as the prevention paradox [17]. This observation supports the need for interventions targeting all gamblers, not only those at high risk.

In Japan, the widespread availability of gambling, with most gamblers falling into the non- and low-risk categories [11], suggests that particular attention should be paid to the prevention paradox. The Japanese government has increased social awareness of gambling disorders based on *The Basic Act on Measures Against Gambling Addiction* [18], enacted alongside *the Act on Development of Specified Integrated Resort Districts* [19]. However, this act mainly focuses on preventing and intervening in gambling disorders in individuals rather than addressing GRHs from a public health perspective. Consequently, the approach may promote a misconception that if an individual is not addicted, there are no problems associated with gambling. To facilitate a comprehensive social discussion on the benefits and harms of gambling, understanding the scope and scale of GRHs is necessary. However, no study has examined GRHs in Japan, indicating lack of quantitative evidence on GRHs among Japanese gamblers.

Therefore, this study aimed to estimate the number of Japanese gamblers experiencing GRHs across six life domains and present their distribution by their problem gambling risk level.

## Methods

### Study design

This cross-sectional study relied on an online survey conducted between August 5 and 11, 2020. It examined the prevalence of GRHs experienced by Japanese gamblers between January 1 and December 31, 2019, across four risk levels (non-problem, low-, moderate-, and high-risk gamblers) classified by the Problem Gambling Severity Index (PGSI) [20] through a two-step approach (see Procedure section).

The data were adjusted for population weighting to align with the demographic profile of Japanese gamblers, using information from two nationally representative cross-sectional surveys: *the 2017 Comprehensive Survey of Living Conditions* [21] and *the 2017 Epidemiological Survey on Gambling Addictions* [22].

#### The comprehensive survey of living conditions [21]

The Japanese Ministry of Health, Labor, and Welfare conducts a comprehensive nationwide survey of households and their members every 3 years, supplemented by smaller annual surveys in the interim years. This survey collects data on health, medical care, income, savings, and other various living conditions. In this study, we used demographic data from the 2017 survey (smaller annual survey) on sex, age, and marital status to align with the year in which *the Epidemiological Survey on Gambling Addictions* [22] was conducted.

#### 2017 epidemiological survey on gambling addictions in Japan [22]

*The Basic Act on Measures Against Gambling Addiction* [18], enacted in 2018, mandates that the Japanese government conduct comprehensive surveys every 3 years to ascertain the current landscape of gambling addiction. Before the establishment of this Act in 2017, the National Hospital Organization Kurihama Medical and Addiction Center conducted a nationwide epidemiological survey. This survey aimed to assess gambling addiction in Japan and was commissioned by the Japan Agency for Medical Research and Development.

Individuals were selected through a two-stage random sampling from Basic Resident Registers at 300 locations across Japan. This interview-based survey included demographic questions and screening tests for gambling disorder/problem gambling: the South Oaks Gambling Screen [23], the Problem Gambling Severity Index (PGSI) [20, 24], and the Diagnostic and Statistical Manual of Mental Disorders criteria [9].

In the 2017 survey, out of 5,306 valid respondents to the PGSI questions (valid response rate 53.1%; 2,421 males and 2,884 females; age range 20–74 years), 37.8% (1,145 males and 863 females) reported having gambled in the past year. The breakdown of problem gambling risk among them was as follows: non-risk 31.5%, low-risk 4.0%, moderate-risk 1.8%, and high-risk 0.6%. This study used data on problem gambling risk distribution and demographic data, including sex, age, and marital status.

### Study setting, participants, and sample size

This study adopted an online survey model, referencing methodologies from previous studies [14, 15]. Participants were individuals aged ≥ 20 years engaged in public gambling (horse racing, boat racing, bicycle racing, and motor bicycle racing) and EGMs (Pachinko and Pachi-slot) more than once during 2019. The recruitment was conducted through *Cross Marketing Group, Inc*., a Japanese market research company with over 5 million active survey monitors. The company’s monitors were pre-registered and had participated in an annual screening survey, including questions about whether they engaged in public gambling and EGMs (Pachinko and Pachi-slot). Our online survey participants were gambling monitors who completed this process.

Given that this study was the first to address GRHs in Japan, specific hypotheses were not examined. The final sample size was set at 4000, with approximately 80 participants in each stratum for data adjustment with population weighting (see Statistical Analysis section). Compared to a previous Australian online survey with 1,524 participants [14], this study’s sample size of 4000 was considered sufficient.

### Measurements

#### Demographic information

Data on age (year of birth), sex, marital status, final education, occupation, and annual income, excluding gambling wins as of December 31, 2019, were collected.

#### Gambling activities in 2019

Data on the frequency and time of gambling (per day), a form of gambling with the highest expenditure, and annual losses owing to gambling were collected. The classification of gambling forms followed *the 2017 Epidemiological Survey on Gambling Addictions* [22]: horse racing, bicycle racing, boat racing, motorbike racing, lottery/numbers/scratch, toto (football betting), Pachinko (EGM), Pachi-slot (EGM), gaming with arcade prizes at arcade games, investment (e.g., stock market and transaction currency), overseas casino, and others.

#### Problem gambling risk

The PGSI [20, 24] was employed to determine the severity of the problem gambling risk for the survey participants. The scale comprises nine items that include the diagnostic features and negative consequences of gambling. Each item is scored as follows: never = 0, sometimes = 1, most of the time = 2, and almost always = 3, with a total score ranging from 0 to 27. The cutoffs in *the 2017 Epidemiological Survey on Gambling Addiction* [22] were adopted for this study. However, the risk of problem gambling was viewed as a spectrum, defined as follows: 0 = non-risk gamblers (NRGs), 1–2 = low-risk gamblers (LRGs), 3–7 = moderate-risk gamblers (MRGs), 8–27 = high-risk gamblers (HRGs). The prevalence of HRGs is a commonly used indicator in epidemiological surveys worldwide, reflecting the prevalence of gambling disorders [10]. The Japanese version of the PGSI has been verified for its reliability and validity [24].

#### Gambling-related harms

To assess the negative experiences caused by gambling, we used a 72-item questionnaire of GRHs [12, 14]. The 72 items of the GRHs were categorized into six domains: financial (14 items); relationships (10 items); emotional/psychological (10 items); health (16 items); work/study (10 items); and others in daily lives (12 items), including various levels of severity. For instance, *“reduction of savings”* falls under mild financial harm, and *“attempted suicide”* is one of the most severe indicators of emotional/psychological harm. All questions had binary answers: i.e., respondents indicated whether or they had experienced each specific harm due to gambling in 2019. The questionnaire was translated into Japanese by the authors and modified to fit Japan’s cultural and social context based on the opinions of gamblers, their family members, and service providers for gamblers. Additionally, the questionnaire was back-translated and validated by one of the questionnaire developers, a co-author (MB). The validity and reliability of the original scale have been previously verified [14]. The Cronbach’s alpha coefficient for this study was 0.95.

### Procedure

Survey invitations were emailed to randomly selected individuals, based on gambling monitors’ demographic data, between August 5 and 10, 2020.

The survey comprised two steps. The preliminary survey was conducted to create 48 participant strata for population weighting adjustments, gathering data on basic demographics, 2019 gambling activities, and the PGSI. Subsequently, the main survey collected data on the GRH 72 items. Invitations were sent to the potential participants of the main survey in six installments until the target sample size was reached. Participants were rewarded with points exchangeable for cash as per *Cross Marketing Group, Inc*. regulations.

### Statistical analysis

#### Descriptive analysis of participant characteristics in the main survey

The socio-demographic data and gambling activity of the survey participants by the PGSI severity were described to confirm the participants’ characteristics.

#### Population weighting of data on the GRH 72 items

The online survey data on GRH were statistically adjusted to reflect the demographic profile of Japanese gamblers. Forty-eight strata were set based on the following variables. These variables were selected because male sex and young age are well-established as risk factors for gambling disorder [25], and the GRH questionnaire includes harms regarding an individual’s life stage and social and family roles.

1. Sex (2 categories: Male, Female).
2. Age (3 categories: 20–39, 40–59, ≥ 60 years).
3. Marital Status (2 categories: Married/In Partnership, Single/Bereaved/Separated/Divorced).
4. PGSI Severity (4 categories: Non-risk, Low-risk, Moderate-risk, High-risk).

The prevalence of each of the GRH 72 items was calculated across these 48 strata. Using data from *the 2017 Comprehensive Survey of Living Conditions* [21] and *the 2017 Epidemiological Survey on Gambling Addictions* [22], the population of Japanese gamblers for each stratum was estimated (Additional Table 1). The prevalence of the 72 GRHs within each stratum was subsequently multiplied by the respective stratum’s population estimate to determine the number of Japanese gamblers experiencing GRH. These figures were subsequently aggregated according to each PGSI severity and for the population as whole. Finally, we calculated the gamblers’ proportion of each PGSI severity relative to the total.

#### Summary of nationwide aggregate and distribution of gamblers who experienced GRH across six life domains

We summarized the distribution of those GRHs by PGSI severity across the six life domains. The estimated number of gamblers who experienced one or more GRH(s) in each domain was visualized in a mosaic plot. Although prior studies have presented mosaic plots of the total number of harms across the six life domains [14, 16], we used this method because the number of harms in each domain varied, and a single gambling experience might be counted multiple times through similar harms.

All the statistical analyses were performed using JMP Pro16 (SAS Institute Inc. Cary, NC, USA), and the mosaic plots were created using R software [26].

### Ethics

This study was approved by the Research Ethics Committee of Otani University (Approval No. 020 − 01, dated July 8, 2020) and the Medical Research Ethics Committee of Kyoto University Medical School (Approval No. R2582, dated July 28, 2020). In compliance with the Declaration of Helsinki principles, detailed information about the study—including its objectives, procedures, participant incentives, potential psychological risks, and data confidentiality measures—was provided on the initial webpage to all visitors. Only participants who provided informed consent were permitted to access the questionnaire.

## Results

### Participant characteristics in the main survey

**Fig. 1:**
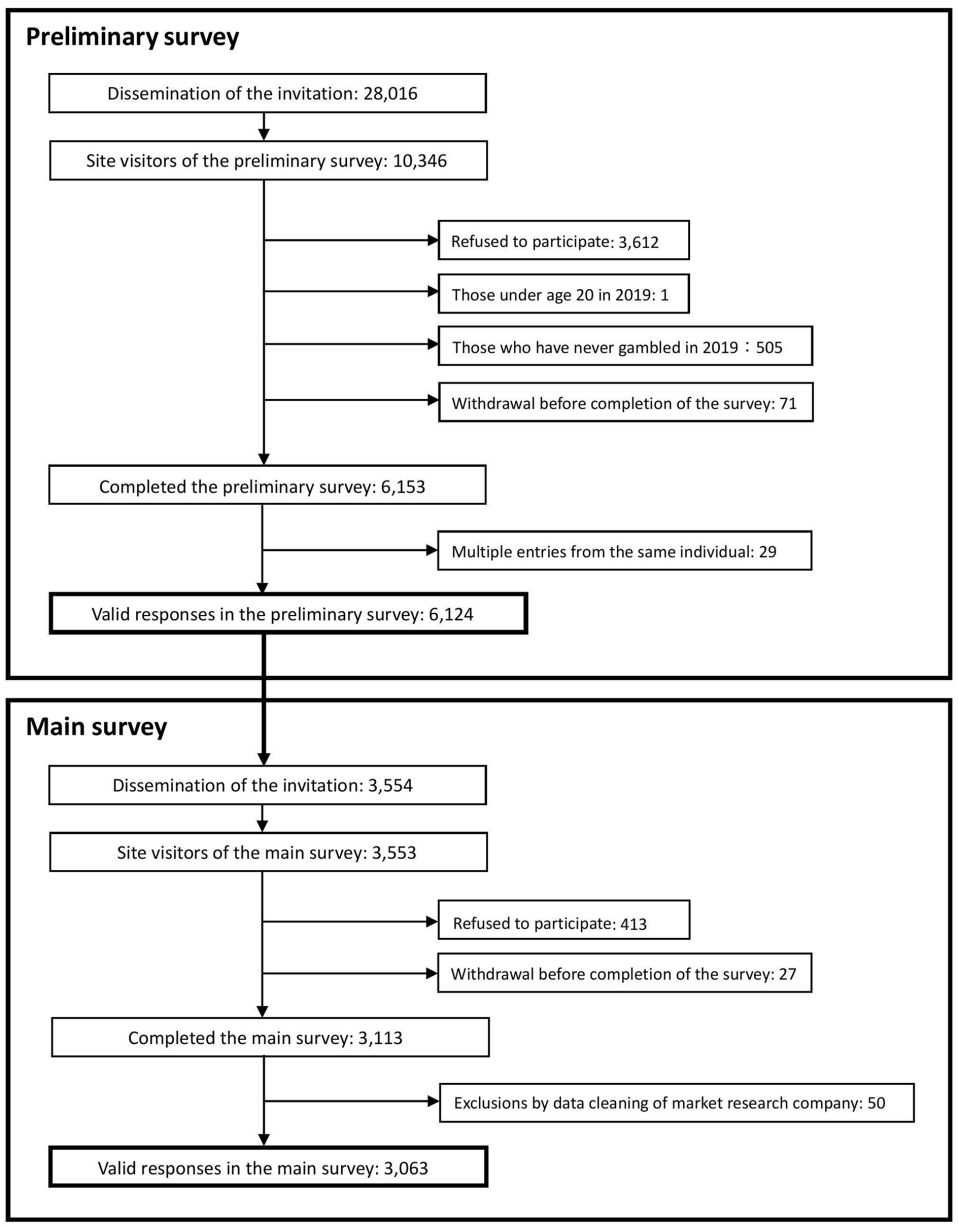
Participant selection flowchart

Of the 28,016 gambling monitors invited, 10,346 accessed the survey site, and 6,124 completed the preliminary survey. Among these, 3,554 were eligible for the main survey, of whom 3,063 provided valid responses (Fig. 1). In the main survey, 52.7% of participants (*n* = 1,614) were male, with a mean age of 49.8 years in 2019 (SD = 13.4, median = 50, range = 20–87). Wide variation in sample sizes across participant strata was observed, ranging from 12 to 100 (Additional Table 1).

Table 1 presents the basic demographic data of these participants. Among the participants, 46.7% had graduated from a university or graduate school, and 33.3% reported an annual income of less than 2 million yen. Table 2 shows the participants’ gambling activities in 2019, overall and categorized by PGSI severity. The activities involving the highest expenditures were horse racing, Pachinko, Pachi-slot, and lotteries.

**Table 1:** Socio-demographic characteristics of the online survey participants by PGSI severity

	Overall		Non-risk (PGSI: 0)		Low-risk (PGSI: 1–2)		Moderate-risk (PGSI: 3–7)		High-risk (PGSI: 8–27)	
	*N* = 3,063		*n* = 782		*n* = 593		*n* = 911		*n* = 777	
**Employment status**										
Self-employed, freelancer *^a^	310	(10.1%)	104	(13.3%)	52	(8.8%)	91	(10.0%)	63	(8.1%)
Regular employment	1,322	(43.2%)	300	(38.4%)	242	(40.8%)	395	(43.4%)	385	(49.5%)
Part-time employee *^b^	611	(19.9%)	153	(19.6%)	133	(22.4%)	169	(18.6%)	156	(20.1%)
Student	23	(0.8%)	5	(0.6%)	3	(0.5%)	9	(1.0%)	6	(0.8%)
House duties	328	(10.7%)	92	(11.8%)	63	(10.6%)	119	(13.1%)	54	(6.9%)
Unemployed	460	(15.0%)	125	(16.0%)	98	(16.5%)	124	(13.6%)	113	(14.5%)
Others	9	(0.3%)	3	(0.4%)	2	(0.3%)	4	(0.4%)	0	(0.0%)
**Final education**										
No schooling	35	(1.1%)	5	(0.6%)	6	(1.0%)	7	(0.8%)	17	(2.2%)
Junior high school	91	(3.0%)	13	(1.7%)	18	(3.0%)	23	(2.5%)	37	(4.8%)
High school	987	(32.2%)	247	(31.6%)	186	(31.4%)	306	(33.6%)	248	(31.9%)
Junior/technical college	520	(17.0%)	141	(18.0%)	101	(17.0%)	164	(18.0%)	114	(14.7%)
University or Graduate school	1,427	(46.6%)	376	(48.1%)	281	(47.4%)	410	(45.0%)	360	(46.3%)
Invalid answer	3	(0.1%)	0	(0.0%)	1	(0.2%)	1	(0.1%)	1	(0.1%)
**Personal annual income (JPN): 1,000**										
Nil income or less than 2,000	1,019	(33.3%)	280	(35.8%)	192	(32.4%)	315	(34.6%)	232	(29.9%)
2,000-less than 4,000	897	(29.3%)	219	(28.0%)	176	(29.7%)	267	(29.3%)	235	(30.2%)
4,000-less than 6,000	594	(19.4%)	143	(18.3%)	113	(19.1%)	177	(19.4%)	161	(20.7%)
6,000-less than 8,000	322	(10.5%)	76	(9.7%)	69	(11.6%)	91	(10.0%)	86	(11.1%)
8,000 or more	231	(7.5%)	64	(8.2%)	43	(7.3%)	61	(6.7%)	63	(8.1%)

PGSI: Problem Gambling Severity Index

JPN: Japanese Yen

*^a^ Self-employed includes family employees

*^b^ Part-time employees include contracts and dispatches

**Table 2:** Gambling activity among the online main survey participants by PGSI severity

	Overall		Non-risk (PGSI: 0)		Low-risk (PGSI: 1–2)		Moderate-risk (PGSI: 3–7)		High-risk (PGSI: 8–27)	
	*N* = 3,063		*n* = 782		*n* = 593		*n* = 911		*n* = 777	
**Gambling frequency**										
Less than 1/month	637	(20.8%)	293	(37.5%)	134	(22.6%)	162	(17.8%)	48	(6.2%)
1–3/month	871	(28.4%)	215	(27.5%)	203	(34.2%)	274	(30.1%)	179	(23.0%)
1–2/week	983	(32.1%)	205	(26.2%)	193	(32.5%)	324	(35.6%)	261	(33.6%)
3–4/week	144	(4.7%)	14	(1.8%)	17	(2.9%)	38	(4.2%)	75	(9.7%)
5–6/week	318	(10.4%)	41	(5.2%)	42	(7.1%)	98	(10.8%)	137	(17.6%)
Every day	110	(3.6%)	14	(1.8%)	4	(0.7%)	15	(1.6%)	77	(9.9%)
**Forms of gambling with the most money spent**										
Pachinko (EGM)	806	(26.3%)	108	(13.8%)	163	(27.5%)	250	(27.4%)	285	(36.7%)
Pachi-slot (EGM)	420	(13.7%)	46	(5.9%)	70	(11.8%)	137	(15.0%)	167	(21.5%)
Horse racing	967	(31.6%)	316	(40.4%)	207	(34.9%)	284	(31.2%)	160	(20.6%)
Bicycle racing	43	(1.4%)	7	(0.9%)	10	(1.7%)	8	(0.9%)	18	(2.3%)
Boat racing	104	(3.4%)	25	(3.2%)	12	(2.0%)	32	(3.5%)	35	(4.5%)
Motorbike racing	16	(0.5%)	5	(0.6%)	3	(0.5%)	6	(0.7%)	2	(0.3%)
Gaming	20	(0.7%)	6	(0.8%)	4	(0.7%)	5	(0.5%)	5	(0.6%)
Offshore online gambling	4	(0.1%)	0	(0.0%)	0	(0.0%)	0	(0.0%)	4	(0.5%)
Land-based casino abroad	11	(0.4%)	1	(0.1%)	2	(0.3%)	6	(0.7%)	2	(0.3%)
Lottery, populations, scratch cards	506	(16.5%)	218	(27.9%)	92	(15.5%)	134	(14.7%)	62	(8.0%)
Toto (football betting)	81	(2.6%)	32	(4.1%)	17	(2.9%)	19	(2.1%)	13	(1.7%)
Stock market	80	(2.6%)	17	(2.2%)	13	(2.2%)	27	(3.0%)	23	(3.0%)
Transaction currency	5	(0.2%)	1	(0.1%)	0	(0.0%)	3	(0.3%)	1	(0.1%)
**Annual loss due to gambling (JPN)**										
Mean	557,422		46,070		83,301		123,074		1,943,166	
SD	18,365,993		197,804		247,714		319,251		364,444	
Median	30,000		10,000		24,000		35,000		100,000	
Range	0–1,000,000,000		0–5,000,000		0–3,500,000		0–6,000,000		0–1,000,000,000	
**Gambling time per day (minutes)**										
Mean	161		102		145		167		224	
SD	149		124		126		148		163	
Median	120		60		120		150		180	
Range	1–1,440		1–1,210		1–796		1–1,440		1–1,215	

PGSI: Problem Gambling Severity Index

JPN: Japanese Yen

### Nationwide aggregate and distribution of gamblers who experienced GRHs in Japan

Drawing data from *the 2017 Comprehensive Survey of Living Conditions* [21] and *the 2017 Epidemiological Survey on Gambling Addictions* [22], it was estimated that 38,991,500 (30.8%) out of 126.8 million Japanese residents had gambled at least once in 2019. Their distribution across the PGSI severity was 32,331,900 NRGs, 4,245,100 LRGs, 1,840,700 MRGs, and 573,900 HRGs.

**Fig. 2:**
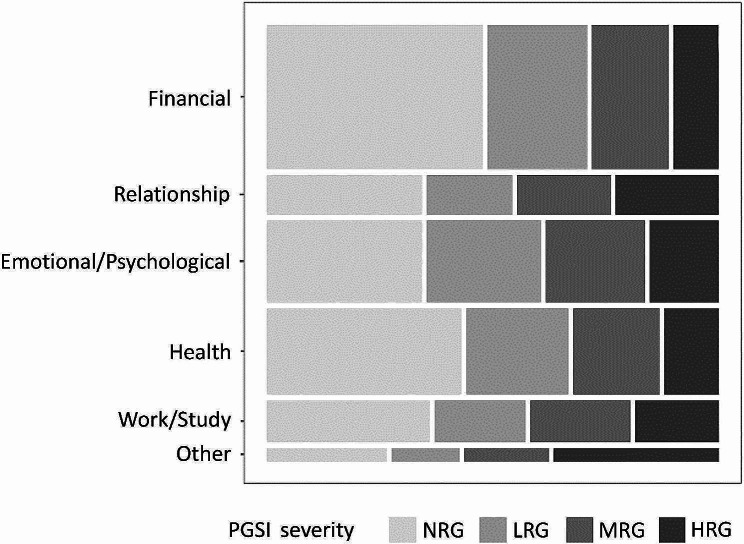
Distribution of estimated Japanese gamblers with at least one harm in each domain by PGSI severity PGSI: Problem Gambling Severity Index; NRG: non-risk gamblers; LRG: low-risk gamblers; MRG: moderate-risk gamblers; HRG: high-risk gamblers

The average number of GRHs per person increased with more severe PGSI risk levels: 0.3 for NRGs, 0.9 for LRGs, 2.1 for MRGs, and 9.1 for HRGs (see Additional Table 2). The data obtained through the online survey were weighted using those of the national surveys. The mosaic plot in Fig. 2 visualizes the distribution of the estimated number of Japanese gamblers who experienced at least one GRH in each domain in 2019, categorized by PGSI severity. The estimated numbers in descending order for the six domains (with the proportion of each PGSI to overall) were as follows: financial harms at 4,438,300 (NRG 49.3%, LRG 22.8%, MRG 17.6%, HRG 10.4%), health harms at 2,702,900 (NRG 44.4%, LRG 23.4%, MRG 19.7%, HRG 12.5%), emotional/psychological harms at 2,536,000 (NRG 35.4%, LRG 26.1%, MRG 22.7%, HRG 15.8%); work/study harms at 1,306,600 (NRG 37.2%, LRG 20.8%, MRG 22.9%, HRG 19.1%); relationship harms at 1,275,900 (NRG 35.4%, LRG 19.6%, MRG 21.4%, HRG 23.6%); and other harms at 457,500 (NRG 27.4%, LRG 15.6%, MRG 19.4%, HRG 37.6%) (see Additional Table 3). The NRGs and LRGs accounted for more than half of the gamblers overall across the five life domains, except for ‘other harms,’ at the population level.

### Detailed analysis of specific GRHs

Tables 3, 4, 5, 6, 7 and 8 present the estimated population and distribution of gamblers who experienced specific GRHs, categorized by PGSI severity. The prevalence of GRHs tends to increase with higher PGSI severity (see Additional Tables 4, 5, 6, 7, 8 and 9). At the individual level, HRGs reported significantly more severe harms, such as *“need of emergency or temporary accommodation”* (Table 3), *“attempted suicide”* (Table 6), *“lost job”* (Table 7), and *“committing a crime or stealing to fund gambling”* (Table 8). Although less frequent, NRGs and LRGs also reported serious harms, including *“bankruptcy”* (Table 3), *“actual separation or ending a relationship”* (Table 4), *“excluded from study”* (Table 7), and *“experiences with violence”* (Table 8).

**Table 3:** Estimated population and distribution by PGSI severity of gamblers who experienced financial harm in 2019

	Japanese gamblers who experienced harm *N* = 38,991,500 (prevalence)		Non-risk (PGSI: 0) *n* = 32,331,900		Low-risk (PGSI: 1–2) *n* = 4,245,100		Moderate-risk (PGSI: 3–7) *n* = 1,840,700		High-risk (PGSI: 8–27) *n* = 573,900	
**Financial harms**										
Reduction of my savings	3,339,000	(8.6%)	1,819,700	(54.5%)	620,500	(18.6%)	560,800	(16.8%)	338,000	(10.1%)
Reduction of my available spending money	1,019,100	(2.6%)	248,600	(24.4%)	280,700	(27.5%)	254,500	(25.0%)	235,300	(23.1%)
Increased credit card debt	186,300	(0.5%)	0	(0.0%)	25,400	(13.6%)	50,600	(27.2%)	110,300	(59.2%)
Sold personal items	172,500	(0.4%)	0	(0.0%)	7,400	(4.3%)	62,700	(36.3%)	102,400	(59.4%)
Took on additional employment	79,500	(0.2%)	0	(0.0%)	17,600	(22.2%)	17,600	(22.1%)	44,300	(55.7%)
Late payments on bills (e.g., utilities, rates)	93,400	(0.2%)	39,500	(42.3%)	0	(0.0%)	8,900	(9.5%)	45,000	(48.2%)
Less spending on recreational expenses such as eating out, going to movies, or other entertainment	574,500	(1.5%)	185,100	(32.2%)	144,000	(25.1%)	164,000	(28.5%)	81,300	(14.2%)
Less spending on beneficial expenses such as insurance, education, car, and home maintenance	80,200	(0.2%)	19,500	(24.3%)	300	(0.3%)	12,600	(15.7%)	47,900	(59.6%)
Less spending on essential expenses such as medications, healthcare, and food	146,100	(0.4%)	20,400	(14.0%)	11,500	(7.9%)	47,600	(32.5%)	66,600	(45.6%)
Needed assistance from welfare organizations (foodbanks or emergency bill payments)	64,200	(0.2%)	19,500	(30.4%)	0	(0.0%)	0	(0.0%)	44,700	(69.6%)
Loss of supply of utilities (e.g., electricity, gas)	44,700	(0.1%)	0	(0.0%)	0	(0.0%)	2,500	(5.6%)	42,200	(94.4%)
Loss of significant assets (e.g., car, home, business, superannuation)	55,400	(0.1%)	0	(0.0%)	300	(0.5%)	4,000	(7.2%)	51,200	(92.3%)
Bankruptcy	89,400	(0.2%)	59,000	(66.0%)	0	(0.0%)	5,800	(6.4%)	24,700	(27.6%)
Needed emergency or temporary accommodation	19,200	(0.0%)	0	(0.0%)	0	(0.0%)	0	(0.0%)	19,200	(100.0%)

PGSI: Problem Gambling Severity Index

**Table 4:** Estimated population and distribution by PGSI severity of gamblers who experienced relationship harm in 2019

	Japanese gamblers who experienced harm *N* = 38,991,500 (prevalence)		Non-risk (PGSI: 0) *n* = 32,331,900		Low-risk (PGSI: 1–2) *n* = 4,245,100		Moderate-risk (PGSI: 3–7) *n* = 1,840,700		High-risk (PGSI: 8–27) *n* = 573,900	
**Relationship harms**										
Spent less time with people I care about	613,400	(1.6%)	180,500	(29.4%)	132,400	(21.6%)	142,400	(23.2%)	158,200	(25.8%)
Got less enjoyment from time spent with people I care about	260,200	(0.7%)	42,300	(16.3%)	48,600	(18.7%)	62,800	(24.1%)	106,500	(40.9%)
Neglected my relationship responsibilities	109,400	(0.3%)	0	(0.0%)	5,400	(4.9%)	18,500	(16.9%)	85,500	(78.2%)
Spent less time attending social events (non-gambling related)	317,300	(0.8%)	134,700	(42.5%)	34,100	(10.8%)	55,400	(17.4%)	93,100	(29.3%)
Experienced greater tension in my relationships (suspicion, lying, resentment)	84,600	(0.2%)	19,500	(23.1%)	0	(0.0%)	9,500	(11.2%)	55,600	(65.7%)
Experienced greater conflict in my relationships (arguing, fighting, ultimatums)	39,900	(0.1%)	0	(0.0%)	1,800	(4.5%)	2,800	(7.1%)	35,300	(88.4%)
Felt belittled in my relationships	89,900	(0.2%)	0	(0.0%)	5,000	(5.5%)	30,300	(33.7%)	54,600	(60.7%)
Threat of separation or ending a relationship/ relationships	140,900	(0.4%)	21,800	(15.5%)	5,400	(3.8%)	22,600	(16.1%)	91,000	(64.6%)
Actual separation or ending of a relationship	79,100	(0.2%)	19,500	(24.7%)	1,800	(2.3%)	700	(0.9%)	57,000	(72.1%)
Social isolation (felt excluded or shut-off from others)	85,200	(0.2%)	19,500	(22.9%)	0	(0.0%)	0	(0.0%)	65,700	(77.1%)

PGSI: Problem Gambling Severity Index

**Table 5:** Estimated population and distribution by PGSI severity of gamblers who experienced emotional/psychological harm in 2019

	Japanese gamblers who experienced harm *N* = 38,991,500 (prevalence)		Non-risk (PGSI: 0) *n* = 32,331,900		Low-risk (PGSI: 1–2) *n* = 4,245,100		Moderate-risk (PGSI: 3–7) *n* = 1,840,700		High-risk (PGSI: 8–27) *n* = 573,900	
**Emotional/psychological harms**										
Felt distressed about my gambling	419,500	(1.1%)	60,300	(14.4%)	78,300	(18.7%)	105,600	(25.2%)	175,300	(41.8%)
Felt ashamed of my gambling	348,600	(0.9%)	62,200	(17.8%)	57,300	(16.4%)	89,800	(25.8%)	139,300	(40.0%)
Felt like failure	499,900	(1.3%)	162,600	(32.5%)	69,000	(13.8%)	104,700	(21.0%)	163,600	(32.7%)
Felt insecure or vulnerable	521,000	(1.3%)	99,600	(19.1%)	117,200	(22.5%)	162,000	(31.1%)	142,100	(27.3%)
Felt angry about not controlling my gambling	292,500	(0.8%)	85,700	(29.3%)	19,800	(6.8%)	73,200	(25.0%)	113,900	(38.9%)
Felt worthless	262,000	(0.7%)	79,800	(30.5%)	54,100	(20.7%)	54,900	(21.0%)	73,100	(27.9%)
Had regrets that made me feel sorry about my gambling	805,900	(2.1%)	193,800	(24.0%)	179,200	(22.2%)	258,400	(32.1%)	174,500	(21.7%)
Feelings of hopelessness about gambling	1,108,500	(2.8%)	470,900	(42.5%)	243,600	(22.0%)	228,700	(20.6%)	165,300	(14.9%)
Feeling of extreme distress	276,000	(0.7%)	78,600	(28.5%)	19,800	(7.2%)	37,900	(13.7%)	139,700	(50.6%)
Thoughts of running away or escaping	320,900	(0.8%)	143,100	(44.6%)	19,200	(6.0%)	47,700	(14.9%)	111,000	(34.6%)

PGSI: Problem Gambling Severity Index

**Table 6:** Estimated population and distribution by PGSI severity of gamblers who experienced health harm in 2019

	Japanese gamblers who experienced harm *N* = 38,991,500 (prevalence)		Non-risk (PGSI: 0) *n* = 32,331,900		Low-risk (PGSI: 1–2) *n* = 4,245,100		Moderate-risk (PGSI: 3–7) *n* = 1,840,700		High-risk (PGSI: 8–27) *n* = 573,900	
**Health harms**										
Reduced physical activity due to my gambling	634,400	(1.6%)	254,400	(40.1%)	139,000	(21.9%)	120,900	(19.1%)	120,100	(18.9%)
Stress-related health problems (e.g., high blood pressure headaches)	218,900	(0.6%)	41,400	(18.9%)	42,500	(19.4%)	48,400	(22.1%)	86,500	(39.5%)
Loss of sleep due to spending time gambling	181,000	(0.5%)	19,500	(10.8%)	22,700	(12.5%)	67,300	(37.2%)	71,600	(39.5%)
Loss of sleep due to stress or worry about gambling or gambling	153,700	(0.4%)	0	(0.0%)	15,200	(9.9%)	37,800	(24.6%)	100,700	(65.5%)
Neglected my hygiene and self-care	157,000	(0.4%)	19,500	(12.4%)	42,800	(27.3%)	23,100	(14.7%)	71,600	(45.6%)
Neglected my medical needs (including taking prescribed medications)	35,800	(0.1%)	0	(0.0%)	0	(0.0%)	5,400	(15.2%)	30,300	(84.8%)
Did not eat as much or as often as I should	417,100	(1.1%)	187,900	(45.0%)	71,800	(17.2%)	73,500	(17.6%)	83,900	(20.1%)
Ate too much	284,300	(0.7%)	176,300	(62.0%)	23,000	(8.1%)	42,600	(15.0%)	42,300	(14.9%)
Increased my use of tobacco	1,346,100	(3.5%)	691,300	(51.4%)	288,100	(21.4%)	248,600	(18.5%)	118,000	(8.8%)
Increased my consumption of alcohol	287,600	(0.7%)	127,700	(44.4%)	17,000	(5.9%)	86,900	(30.2%)	56,100	(19.5%)
Increased experience of depression	56,500	(0.1%)	18,900	(33.5%)	1,800	(3.2%)	3,400	(6.1%)	32,300	(57.2%)
Increased use of health services due to health issues caused or exacerbated by my gambling	17,100	(0.0%)	0	(0.0%)	0	(0.0%)	0	(0.0%)	17,100	(100.0%)
Committed acts of self-harm	13,500	(0.0%)	0	(0.0%)	0	(0.0%)	900	(6.7%)	12,600	(93.3%)
Unhygienic living conditions (e.g., living rough, neglected, or unclean housing)	19,200	(0.0%)	0	(0.0%)	0	(0.0%)	0	(0.0%)	19,200	(100.0%)
Required emergency medical treatment for health issues caused or exacerbated by gambling	86,800	(0.2%)	19.500	(22.5%)	0	(0.0%)	0	(0.0%)	67,300	(77.5%)
Attempted suicide	19,900	(0.1%)	0	(0.0%)	1,800	(9.1%)	0	(0.0%)	18,100	(90.9%)

PGSI: Problem Gambling Severity Index

**Table 7:** Estimated population and distribution by PGSI severity of gamblers who experienced work/study harm in 2019

	Japanese gamblers who experienced harm *N* = 38,991,500 (prevalence)		Non-risk (PGSI: 0) *n* = 32,331,900		Low-risk (PGSI: 1-–2) *n* = 4,245,100		Moderate-risk (PGSI: 3–7) *n* = 1,840,700		High-risk (PGSI: 8–27) *n* = 573,900	
**Work/ study harms**										
Reduced performance at work or study (e.g., due to tiredness or distraction)	480,500	(1.2%)	196,700	(40.9%)	60,900	(12.7%)	97,600	(20.3%)	125,300	(26.1%)
Was late for work or study	206,600	(0.5%)	113,500	(54.9%)	14,400	(7.0%)	12,900	(6.2%)	65,800	(31.9%)
Was absent from work or study	297,200	(0.8%)	126,700	(42.6%)	48,100	(16.2%)	47,800	(16.1%)	74,700	(25.1%)
Hindered my job-seeking efforts	36,000	(0.1%)	0	(0.0%)	0	(0.0%)	0	(0.0%)	36.,000	(100.0%)
Used my work or study time to gamble	387,400	(1.0%)	109,900	(28.4%)	107,000	(27.6%)	95,900	(24.8%)	74,600	(19.3%)
Used my work or study resources to gamble	117,400	(0.3%)	0	(0.0%)	28,300	(24.1%)	32,300	(27.5%)	56,800	(48.4%)
Lack of progression in my job or study	150,000	(0.4%)	53,600	(35.8%)	22,800	(15.2%)	35,700	(23.8%)	37,800	(25.2%)
Conflict with my colleagues	25,900	(0.1%)	0	(0.0%)	0	(0.0%)	1,300	(4.9%)	24,600	(95.1%)
Lost my job	47,700	(0.1%)	0	(0.0%)	0	(0.0%)	0	(0.0%)	47,700	(100.0%)
Excluded from study	45,200	(0.1%)	19,500	(43.1%)	4,500	(10.0%)	0	(0.0%)	21,200	(46.9%)

PGSI: Problem Gambling Severity Index

**Table 8:** Estimated population and distribution by PGSI severity of gamblers who experienced other harm in 2019

	Japanese gamblers who experienced harm *N* = 38,991,500 (prevalence)		Non-risk (PGSI: 0) *n* = 32,331,900		Low-risk (PGSI: 1–2) *n* = 4,245,100		Moderate-risk (PGSI: 3–7) *n* = 1,840,700		High-risk (PGSI: 8–27) *n* = 573,900	
**Other harms**										
Left children unsupervised	49,400	(0.1%)	0	(0.0%)	0	(0.0%)	13,500	(24.7%)	35,900	(72.6%)
Did not fully attend to the needs of children	195,600	(0.5%)	0	(0.0%)	28,100	(14.4%)	83,900	(42.9%)	83,600	(42.7%)
Took money or items from friends or family without asking first	91,900	(0.2%)	19,500	(21.2%)	5,400	(5.8%)	9,300	(10.2%)	57,600	(62.8%)
Promised to pay back money without genuinely intending to do so	47,200	(0.1%)	0	(0.0%)	0	(0.0%)	0	(0.0%)	47,200	(100.0%)
Arrested for unsafe driving	16,100	(0.0%)	0	(0.0%)	0	(0.0%)	0	(0.0%)	16,100	(100.0%)
Reduced my contribution to religious or cultural practices	33,000	(0.1%)	0	(0.0%)	0	(0.0%)	200	(0.6%)	32,800	(99.4%)
Felt less connected to my religious or cultural community	140,500	(0.4%)	40,300	(28.7%)	21,000	(14.9%)	37,100	(26.4%)	42,000	(29.9%)
Felt that I had shamed my family name within my religious or cultural community	33,700	(0.1%)	0	(0.0%)	1,800	(5.4%)	6,100	(18.0%)	25,800	(76.6%)
Petty theft or dishonesty with respect to government, businesses, or other people	70,900	(0.2%)	19,500	(27.5%)	5,400	(7.6%)	5,400	(7.6%)	40,700	(57.4%)
Felt compelled or forced to commit a crime or steal to fund gambling	35,100	(0.1%)	0	(0.0%)	0	(0.0%)	900	(2.6%)	34,200	(97.4%)
Outcast from religious or cultural community due to involvement with gambling	44,800	(0.1%)	0	(0.0%)	0	(0.0%)	4,500	(10.0%)	40,300	(90.0%)
Had experiences with violence (including family/domestic violence)	93,900	(0.2%)	81,300	(86.5%)	0	(0.0%)	200	(0.2%)	12,500	(13.3%)

PGSI: Problem Gambling Severity Index

At the population level, the primary harms experienced by a large segment of gamblers were *“reduction of savings”*, *“reduction of available spending money”*, and *“less spending on recreational expenses”* as financial harms (Table 3); *“spending less time with people cared about”* as relationship harms (Table 4); *“increased use of tobacco”* and *“reduced physical activity owing to gambling”* as health harms (Table 6); *“feelings of hopelessness about gambling,”* and *“had regrets that made them feel sorry about gambling”* as emotional/psychological harms (Table 5); and *“reduced performance at work or study”* as work/study harms (Table 7). NRGs and LRGs accounted for a significant proportion of each harm category, supporting the proposition that the prevention paradox applies to GRH.

## Discussion

To our knowledge, this study is the first in Japan to explore GRHs as experienced by gamblers. Adopting a public health approach, it examines the broader negative consequences associated with gambling in society rather than focusing on the individual pathology of gambling disorder. Among the 126.8 million residents in Japan, an estimated 39.0 million (30.8%) engaged in gambling at least once in 2019. Of them, the financial domain was the most harmed, experienced by 4,438,000, followed by health at 2,703,000, and emotional/psychological at 2,536,000. Notably, the NRGs and LRGs accounted for over 60% of the people who experienced the harm. These results indicate the presence of a prevention paradox in gambling in Japan.

Compared with this study, the domains in which the most frequently experienced harms in Australia [14] and Finland [16], were financial, emotional/psychological, and health, in that order. The variations in the GRH size in each domain between those and the present studies may be attributed to differences in sampling methods, problem gambling risk measurements, statistical analyses, and cultural contexts related to gambling. However, overall, the distribution of GRHs across the six life domains was similar, indicating that financial, emotional/psychological, and health harms are commonly experienced even by NRGs or LRGs.

Out of the GRH 72 items, *“reduction of savings”* and *“reduction of available spending money”* in the financial domain were the most commonly experienced harms among gamblers. These somewhat less severe impacts most clearly illustrate the prevention paradox. Such financial harms were also prevalent in Australian and Finnish studies [14, 16]. Despite some criticisms that financial losses from gambling are merely opportunity costs [27], the participants reported these experiences as negative consequences attributed to gambling, and previous research has demonstrated that they are substantial harms beyond simple costs [28]. The returns from gambling are uncertain and have higher risks than other leisure activities, as gambling is a game of chance involving money. Moreover, the design and system of commercial gambling are noted for leading to expenditures that exceed an individual’s intentions [3, 29]. The results of our study indicate that even NRGs and LRGs can suffer financial harm and encounter financial difficulties as a result of gambling.

The distribution of each type of harm within the health and emotional/psychological domains differed between the present study and previous studies [14, 16]. Specifically, *“increased tobacco use”* emerged as the most prevalent health harm in this study, ranking second among the GRH 72 items. In contrast, *“increased alcohol use”* was more commonly reported in Australia, and *“increased experience of depression”* was in Finland. Additionally, *“hopelessness about gambling”* was identified as the most prevalent emotional/psychological harm in this study, ranking third overall. In Australia and Finland, however, feelings of *“regretting gambling,”* “*feeling like a failure in relation to gambling,”* and *“feeling ashamed of gambling”* were most commonly reported. These differences reflect the variations in social and cultural contexts in each country. For example, in Japan, smoking in Pachinko and Pachi-slot venues was allowed and common until 2020, unlike in Australia, where gambling often involves alcohol consumption in casinos and clubs. The manner in which specific emotions are expressed is also likely to vary across cultures. These findings indicate the variation in how gambling affects individuals’ lifestyles and mental health.

In contrast, the prevention paradox was not observed in severe harms, such as *“needing emergency or temporary accommodation,” “leaving children unsupervised,”* and *“committing a crime or stealing to fund gambling.”* These harms are mainly reported by HRGs and align with clinical problems observed in gambling disorders. However, despite being relatively rare, the NRGs also reported experiences of *“bankruptcy”* or *“violence.”* After adjusting for population weighting, the estimated number of NRGs or LRGs experiencing those severe harms surpassed that of HRGs. Previous studies [14, 16] also revealed severe harms among NRGs and LRGs. A possible explanation for this phenomenon is that the severe harms experienced in 2019 may have resulted from excessive gambling that occurred before that year. A qualitative approach would be useful for exploring the context of serious harms among NRGs.

To mitigate the societal burden of gambling in Japan, policymaking should recognize the prevention paradox in the GRH. Negative consequences of gambling are not exclusive to HRGs and MRGs; NRGs and LRGs, also experienced such consequences. On average, each NRG and LRG experienced less than one harm, which may not warrant a diagnosis of “addiction.” Nevertheless, these “harms” can significantly impact an individual’s well-being and quality of life. Additionally, NRGs and LRGs contributed significantly to the overall harm at the population level. A narrow focus on HRGs or individuals with gambling disorders would underestimate the total social burden of gambling.

Therefore, interventions for gambling issues should broaden their focus beyond controlling gambling disorders to encompass minimizing GRH across society. This comprehensive approach includes maintaining adequate funding for the treatment of gambling disorders, as well as increasing funding for public health measures and wider prevention initiatives. In countries such as Australia and the UK, where GRH is monitored at the state or national level, the approach to addressing gambling issues has transformed from focusing on individual responsible gambling to adopting a public health approach, mandating industry participation [30–33]. This approach emphasizes consumer protection, advocating for the safeguarding of all individuals from GRH risks, regardless of their self-protection ability [34, 35]. Strategies for reducing GRH include curbing excessive gambling consumption through measures such as imposing or increasing entrance fees, regulating addictive EGMs, reducing odds, mandating pre-commitment limits for monetary loss, banning gambling advertisements, and incorporating warnings about GRH into provided gambling information. While initiating epidemiological studies of GRH in Japan is an important first step, it is also necessary to improve the social environment that facilitates easy access to gambling, where EGMs (Pachinko and Pachi-slot) and gambling advertising are widespread.

## Conclusions

In societies with high gambling accessibility, such as Japan, gaining a comprehensive understanding of the multifaceted benefits and harms associated with commercial gambling is critical. This study revealed that a large proportion of Japanese gamblers experiencing GRH are attributed to NRGs and LRGs who would not meet the criteria for gambling disorders. This phenomenon, recognized as the prevention paradox, supports the need for a population-based approach to minimize GRHs across society. Hence, Japanese national strategies for gambling issues should shift from focusing solely on “prevention and intervention of addiction” to a more inclusive aim of “minimizing GRHs” across the entire population. As a first step, conducting extensive investigations and monitoring of GRHs using representative sampling methods is crucial. Furthermore, this approach should encompass the gamblers themselves, their families, and the broader social network.

### Limitations

This study has some limitations. First, the online survey participants were not representative, and the results obtained cannot be generalized to the population with a high degree of confidence, as the participants were recruited through a market research company. Although the study employed a weighted-back adjustment to align the online survey data with the demographic characteristics of Japanese gamblers, potentially important variables might not be included in the stratification. For instance, the proportion of low-income individuals in the online survey sample was greater than that in the general Japanese population. Additionally, while lottery tickets rank as the most engaged gambling activity in Japan, followed by Pachinko, Pachi-slot, and horse racing, in our online survey, those who spent the most money on horse racing were the highest among the participants. Although risk factors for GRH have not yet been well-established, previous studies suggest gambling conditions and social disadvantages as the risk factors [36–38]. Low income level, gambling expenditure being a high proportion of income, high frequency of gambling, certain types of gambling activity, and social disadvantages might be potential risk factors for GRH.

The second limitation pertains to the precision of estimating the social burden of gambling. Recruiting participants for our online survey was challenging in certain demographics, such as women aged 60 and above, resulting in considerable variations in sample size across the 48 strata. Although the study does not present confidence intervals for the estimations, it is important to acknowledge that smaller sample sizes correspond to broader confidence intervals. However, we believe that the impact of these groups on the overall estimated GRHs in Japan would be relatively small, because these groups usually have lower levels of gambling engagement (see Additional Table 1).

Third, this study conducted a web-based survey in August 2020 on gambling experiences during 2019, with all data being self-reported. The lengthy interval between the experiences and the survey—ranging from 8 to 20 months—may have potentially diminished the accuracy of the responses by altering respondents’ memories and perceptions of their past experiences. To validate our findings, future studies should utilize more recent experiential data.

Fourth, there is a temporal discrepancy between the target years of our online survey and the national survey used for population weighting adjustment. Although our study was initially planned to align with *the 2020 Epidemiological Survey on Gambling Addictions* in Japan, we anticipated that the pandemic’s restraint on non-essential outings would influence people’s gambling behavior. Therefore, the target survey year was advanced to 2019, and the 2017 national survey data was employed for population weighting adjustment since understanding GRH under usual conditions would be important for future comparisons.

Finally, this study did not include a survey of harms among family, co-workers and friends of gamblers. According to an Australian study [39], the negative impacts of gambling extend beyond the gamblers themselves, affecting their close associates, including non-gamblers. This is particularly true for close family members when there are shared financial and shared responsibilities. From a public health standpoint, assessing the broader spectrum of GRHs experienced by those in the gambler’s social network is imperative. Therefore, further research should address these limitations by employing representative samples and broadening the scope of the target population.

### Electronic supplementary material

Below is the link to the electronic supplementary material.

Supplementary Material 1

## Data Availability

The datasets of the web survey are available from the corresponding author upon reasonable request.

## Abbreviations

GRH: Gambling-related harm
NRG: Non-risk gambler
LRG: Low-risk gambler
MRG: Moderate-risk gambler
HRG: High-risk gambler
PGSI: Problem Gambling Severity Index
