# Supplementary material for: National burden of gambling in Japan: an estimation from an online-based cross-sectional investigation and national epidemiological survey
Source: BMC Public Health. 2024 Jun 26;24:1703. doi: 10.1186/s12889-024-19197-z (PMC11201868; doi:10.1186/s12889-024-19197-z)
Supplement: Supplementary file 1 — Supplementary Material 1 [file 12889_2024_19197_MOESM1_ESM.pdf]

Additional Table 1. Demographics of Japanese residents and the sample size of the main survey for each stratum

Stratum No.	PGSI	Sex	Age	Marital status	Population of Japanese gamblers unit：1,000 persons	Sample size
**1**	NRG	Male	20-39 y.o.	Spouse, partnership	1876.5	35
**2**	NRG	Male	20-39 y.o.	Never married, bereaved, separated, divorced	1900.8	93
**3**	NRG	Male	40-59 y.o.	Spouse, partnership	5064.7	90
**4**	NRG	Male	40-59 y.o.	Never married, bereaved, separated, divorced	1613.2	40
**5**	NRG	Male	60+ y.o.	Spouse, partnership	5672.9	91
**6**	NRG	Male	60+ y.o.	Never married, bereaved, separated, divorced	1223.4	56
**7**	NRG	Female	20-39 y.o.	Spouse, partnership	1665.5	88
**8**	NRG	Female	20-39 y.o.	Never married, bereaved, separated, divorced	1775.8	91
**9**	NRG	Female	40-59 y.o.	Spouse, partnership	4044.7	90
**10**	NRG	Female	40-59 y.o.	Never married, bereaved, separated, divorced	1263.6	32
**11**	NRG	Female	60+ y.o.	Spouse, partnership	3608.8	59
**12**	NRG	Female	60+ y.o.	Never married, bereaved, separated, divorced	2048.1	17
**13**	LRG	Male	20-39 y.o.	Spouse, partnership	417.0	24
**14**	LRG	Male	20-39 y.o.	Never married, bereaved, separated, divorced	487.4	91
**15**	LRG	Male	40-59 y.o.	Spouse, partnership	841.1	93
**16**	LRG	Male	40-59 y.o.	Never married, bereaved, separated, divorced	438.4	19
**17**	LRG	Male	60+ y.o.	Spouse, partnership	973.7	74
**18**	LRG	Male	60+ y.o.	Never married, bereaved, separated, divorced	359.8	26
**19**	LRG	Female	20-39 y.o.	Spouse, partnership	88.6	49
**20**	LRG	Female	20-39 y.o.	Never married, bereaved, separated, divorced	21.4	83
**21**	LRG	Female	40-59 y.o.	Spouse, partnership	266.7	59
**22**	LRG	Female	40-59 y.o.	Never married, bereaved, separated, divorced	16.8	20
**23**	LRG	Female	60+ y.o.	Spouse, partnership	183.8	39
**24**	LRG	Female	60+ y.o.	Never married, bereaved, separated, divorced	71.9	16
**25**	MRG	Male	20-39 y.o.	Spouse, partnership	303.3	81
**26**	MRG	Male	20-39 y.o.	Never married, bereaved, separated, divorced	414.3	92
**27**	MRG	Male	40-59 y.o.	Spouse, partnership	250.6	92
**28**	MRG	Male	40-59 y.o.	Never married, bereaved, separated, divorced	157.8	47
**29**	MRG	Male	60+ y.o.	Spouse, partnership	254.0	90
**30**	MRG	Male	60+ y.o.	Never married, bereaved, separated, divorced	119.9	57
**31**	MRG	Female	20-39 y.o.	Spouse, partnership	<0.1	89
**32**	MRG	Female	20-39 y.o.	Never married, bereaved, separated, divorced	85.6	95
**33**	MRG	Female	40-59 y.o.	Spouse, partnership	118.5	94
**34**	MRG	Female	40-59 y.o.	Never married, bereaved, separated, divorced	33.7	61
**35**	MRG	Female	60+ y.o.	Spouse, partnership	16.7	91
**36**	MRG	Female	60+ y.o.	Never married, bereaved, separated, divorced	143.7	22
**37**	HRG	Male	20-39 y.o.	Spouse, partnership	75.8	86
**38**	HRG	Male	20-39 y.o.	Never married, bereaved, separated, divorced	97.5	91
**39**	HRG	Male	40-59 y.o.	Spouse, partnership	89.5	90
**40**	HRG	Male	40-59 y.o.	Never married, bereaved, separated, divorced	70.1	53
**41**	HRG	Male	60+ y.o.	Spouse, partnership	127.0	79
**42**	HRG	Male	60+ y.o.	Never married, bereaved, separated, divorced	<0.1	24
**43**	HRG	Female	20-39 y.o.	Spouse, partnership	17.7	100
**44**	HRG	Female	20-39 y.o.	Never married, bereaved, separated, divorced	42.8	91
**45**	HRG	Female	40-59 y.o.	Spouse, partnership	<0.1	57
**46**	HRG	Female	40-59 y.o.	Never married, bereaved, separated, divorced	16.8	36
**47**	HRG	Female	60+ y.o.	Spouse, partnership	<0.1	58
**48**	HRG	Female	60+ y.o.	Never married, bereaved, separated, divorced	71.9	12

PGSI: Problem Gambling Severity Index.
NRG: Non-risk gambler.
LRG: Low-risk gambler.
MRG: Moderate-risk gambler.
HRG: High-risk gambler.
y.o.: years old.

Additional Table 2. Number of gambling-related harm experienced per person by PGSI severity

	**Non-risk PGSI: 0 n=782**	**Low-risk PGSI: 1–2 n=593**	**Moderate-risk PGSI: 3–7 n=911**	**High-risk PGSI: 8–27 n=777**
Mean	0.3	0.9	2.1	9.1
SD	1.0	1.6	3.3	10.3
Median	0	0	1	7
Range	0 –14	0 –12	0 –24	0 –72

PGSI: Problem Gambling Severity Index

Additional Table 3. Estimated number, prevalence and distribution of gamblers experienced at least one GRH in each domain in 2019

	Japanese gamblers who experienced GRH N = 38,991,500	prevalence	Non-risk PGSI: 0 n=32,331,900		Low-risk PGSI: 1–2 n=4,245,100		Moderate-risk PGSI: 3–7 n=1,840,700		High-risk PGSI: 8–27 n=573,900	
Financial harms	4,438,300	11.4%	2,186,200	49.3%	1,010,900	22.8%	781,300	17.6%	459,900	10.4%
Relationship harms	1,275,900	3.3%	451,900	35.4%	250,000	19.6%	273,200	21.4%	300,700	23.6%
Emotional/ psychological harms	2,536,000	6.5%	898,000	35.4%	662,400	26.1%	575,400	22.7%	400,300	15.8%
Health harms	2,702,900	6.9%	1,200,500	44.4%	631,300	23.4%	533,800	19.7%	337,300	12.5%
Work/ study harms	1,306,600	3.4%	485,900	37.2%	271,500	20.8%	299,400	22.9%	249,800	19.1%
Others in daily lives	457,500	1.2%	125,400	27.4%	71,400	15.6%	88,700	19.4%	172,100	37.6%

GRH: Gambling-related harm
PGSI: Problem Gambling Severity Index

Additional Table 4. Estimated prevalence of financial harm in 2019 for Japanese gamblers overall and in each PGSI severity level

	Japanese gamblers overall	Non-risk PGSI: 0	Low-risk PGSI: 1–2	Moderate-risk PGSI: 3–7	High-risk PGSI: 8–27
**Financial harms**					
Reduction of my savings	8.6%	5.6%	14.6%	30.5%	58.9%
Reduction of my available spending money	2.6%	0.8%	6.6%	13.8%	41.0%
Increased credit card debt	0.5%	0.0%	0.6%	2.8%	19.2%
Sold personal items	0.4%	0.0%	0.2%	3.4%	17.8%
Took on additional employment	0.2%	0.0%	0.4%	1.0%	7.7%
Late payments on bills (e.g., utilities, rates)	0.2%	0.1%	0.0%	0.5%	7.8%
Less spending on recreational expenses such as eating out, going to movies, or other entertainment	1.5%	0.6%	3.4%	8.9%	14.2%
Less spending on beneficial expenses such as insurance, education, car, and home maintenance	0.2%	0.1%	0.0%	0.7%	8.3%
Less spending on essential expenses such as medications, healthcare, and food	0.4%	0.1%	0.3%	2.6%	11.6%
Needed assistance from welfare organizations (foodbanks or emergency bill payments)	0.2%	0.1%	0.0%	0.0%	7.8%
Loss of supply of utilities (e.g., electricity, gas)	0.1%	0.0%	0.0%	0.1%	7.3%
Loss of significant assets (e.g., car, home, business, superannuation)	0.1%	0.0%	0.0%	0.2%	8.9%
Bankruptcy	0.2%	0.2%	0.0%	0.3%	4.3%
Needed emergency or temporary accommodation	0.0%	0.0%	0.0%	0.0%	3.4%

PGSI: Problem Gambling Severity Index

Additional Table 5. Estimated prevalence of relationship harm in 2019 for Japanese gamblers overall and in each PGSI severity level

	**Japanese gamblers overall**	**Non-risk PGSI: 0**	**Low-risk PGSI: 1–2**	**Moderate-risk PGSI: 3–7**	**High-risk PGSI: 8–27**
**Relationship harms**					
Spent less time with people I care about	1.6%	0.6%	3.1%	7.7%	27.6%
Got less enjoyment from time spent with people I care about	0.7%	0.1%	1.1%	3.4%	18.6%
Neglected my relationship responsibilities	0.3%	0.0%	0.1%	1.0%	14.9%
Spent less time attending social events (non-gambling related)	0.8%	0.4%	0.8%	3.0%	16.2%
Experienced greater tension in my relationships (suspicion, lying, resentment)	0.2%	0.1%	0.0%	0.5%	9.7%
Experienced greater conflict in my relationships (arguing, fighting, ultimatums)	0.1%	0.0%	0.0%	0.2%	6.2%
Felt belittled in my relationships	0.2%	0.0%	0.1%	1.6%	9.5%
Threat of separation or ending a relationship/ relationships	0.4%	0.1%	0.1%	1.2%	15.9%
Actual separation or ending of a relationship	0.2%	0.1%	0.0%	0.0%	9.9%
Social isolation (felt excluded or shut-off from others)	0.2%	0.1%	0.0%	0.0%	11.4%

PGSI: Problem Gambling Severity Index

Additional Table 6. Estimated prevalence of emotional/ psychological harm in 2019 for Japanese gamblers overall and in each PGSI severity level

	**Japanese gamblers overall**	**Non-risk PGSI: 0**	**Low-risk PGSI: 1–2**	**Moderate-risk PGSI: 3–7**	**High-risk PGSI: 8–27**
**Emotional/psychological harms**					
Felt distressed about my gambling	1.1%	0.2%	1.8%	5.7%	30.5%
Felt ashamed of my gambling	0.9%	0.2%	1.4%	4.9%	24.3%
Felt like failure	1.3%	0.5%	1.6%	5.7%	28.5%
Felt insecure or vulnerable	1.3%	0.3%	2.8%	8.8%	24.8%
Felt angry about not controlling my gambling	0.8%	0.3%	0.5%	4.0%	19.8%
Felt worthless	0.7%	0.2%	1.3%	3.0%	12.7%
Had regrets that made me feel sorry about my gambling	2.1%	0.6%	4.2%	14.0%	30.4%
Feelings of hopelessness about gambling	2.8%	1.5%	5.7%	12.4%	28.8%
Feeling of extreme distress	0.7%	0.2%	0.5%	2.1%	24.3%
Thoughts of running away or escaping	0.8%	0.4%	0.5%	2.6%	19.3%

PGSI: Problem Gambling Severity Index

Additional Table 7. Estimated prevalence of health harm in 2019 for Japanese gamblers overall and in each PGSI severity level

	**Japanese gamblers overall**	**Non-risk PGSI: 0**	**Low-risk PGSI: 1–2**	**Moderate-risk PGSI: 3–7**	**High-risk PGSI: 8–27**
**Health harms**					
Reduced physical activity due to my gambling	1.6%	0.8%	3.3%	6.6%	20.9%
Stress-related health problems (e.g., high blood pressure headaches)	0.6%	0.1%	1.0%	2.6%	15.1%
Loss of sleep due to spending time gambling	0.5%	0.1%	0.5%	3.7%	12.5%
Loss of sleep due to stress or worry about gambling or gambling	0.4%	0.0%	0.4%	2.1%	17.5%
Neglected my hygiene and self-care	0.4%	0.1%	1.0%	1.3%	12.5%
Neglected my medical needs (including taking prescribed medications)	0.1%	0.0%	0.0%	0.3%	5.3%
Did not eat as much or as often as I should	1.1%	0.6%	1.7%	4.0%	14.6%
Ate too much	0.7%	0.5%	0.5%	2.3%	7.4%
Increased my use of tobacco	3.5%	2.1%	6.8%	13.5%	20.6%
Increased my consumption of alcohol	0.7%	0.4%	0.4%	4.7%	9.8%
Increased experience of depression	0.1%	0.1%	0.0%	0.2%	5.6%
Increased use of health services due to health issues caused or exacerbated by my gambling	0.0%	0.0%	0.0%	0.0%	3.0%
Committed acts of self-harm	0.0%	0.0%	0.0%	0.0%	2.2%
Unhygienic living conditions (e.g., living rough, neglected, or unclean housing)	0.0%	0.0%	0.0%	0.0%	3.4%
Required emergency medical treatment for health issues caused or exacerbated by gambling	0.2%	0.1%	0.0%	0.0%	11.7%
Attempted suicide	0.1%	0.0%	0.0%	0.0%	3.2%

PGSI: Problem Gambling Severity Index

Additional Table 8. Estimated prevalence of work/ study harm in 2019 for Japanese gamblers overall and in each PGSI severity level

	**Japanese gamblers overall**	**Non-risk PGSI: 0**	**Low-risk PGSI: 1–2**	**Moderate-risk PGSI: 3–7**	**High-risk PGSI: 8–27**
**Work study harms**					
Reduced performance at work or study (e.g., due to tiredness or distraction)	**1.2%**	0.6%	1.4%	5.3%	21.8%
Was late for work or study	**0.5%**	0.4%	0.3%	0.7%	11.5%
Was absent from work or study	**0.8%**	0.4%	1.1%	2.6%	13.0%
Hindered my job-seeking efforts	**0.1%**	0.0%	0.0%	0.0%	6.3%
Used my work or study time to gamble	**1.0%**	0.3%	2.5%	5.2%	13.0%
Used my work or study resources to gamble	**0.3%**	0.0%	0.7%	1.8%	9.9%
Lack of progression in my job or study	**0.4%**	0.2%	0.5%	1.9%	6.6%
Conflict with my colleagues	**0.1%**	0.0%	0.0%	0.1%	4.3%
Lost my job	**0.1%**	0.0%	0.0%	0.0%	8.3%
Excluded from study	**0.1%**	0.1%	0.1%	0.0%	3.7%

PGSI: Problem Gambling Severity Index

Additional Table 9. Estimated prevalence of other harm in 2019 for Japanese gamblers overall and in each PGSI severity level

	**Japanese gamblers overall**	**Non-risk PGSI: 0**	**Low-risk PGSI: 1–2**	**Moderate-risk PGSI: 3–7**	**High-risk PGSI: 8–27**
**Other harms**					
Left children unsupervised	**0.1%**	0.0%	0.0%	0.7%	6.3%
Did not fully attend to the needs of children	**0.5%**	0.0%	0.7%	4.6%	14.6%
Took money or items from friends or family without asking first	**0.2%**	0.1%	0.1%	0.5%	10.0%
Promised to pay back money without genuinely intending to do so	**0.1%**	0.0%	0.0%	0.0%	8.2%
Arrested for unsafe driving	**0.0%**	0.0%	0.0%	0.0%	2.8%
Reduced my contribution to religious or cultural practices	**0.1%**	0.0%	0.0%	0.0%	5.7%
Felt less connected to my religious or cultural community	**0.4%**	0.1%	0.5%	2.0%	7.3%
Felt that I had shamed my family name within my religious or cultural community	**0.1%**	0.0%	0.0%	0.3%	4.5%
Petty theft or dishonesty with respect to government, businesses, or other people	**0.2%**	0.1%	0.1%	0.3%	7.1%
Felt compelled or forced to commit a crime or steal to fund gambling	**0.1%**	0.0%	0.0%	0.0%	6.0%
Outcast from religious or cultural community due to involvement with gambling	**0.1%**	0.0%	0.0%	0.2%	7.0%
Had experiences with violence (including family/domestic violence)	**0.2%**	0.3%	0.0%	0.0%	2.2%

PGSI: Problem Gambling Severity Index
